# PDHX acetylation facilitates tumor progression by disrupting PDC assembly and activating lactylation-mediated gene expression

**DOI:** 10.1093/procel/pwae052

**Published:** 2024-09-23

**Authors:** Zetan Jiang, Nanchi Xiong, Ronghui Yan, Shi-ting Li, Haiying Liu, Qiankun Mao, Yuchen Sun, Shengqi Shen, Ling Ye, Ping Gao, Pinggen Zhang, Weidong Jia, Huafeng Zhang

**Affiliations:** Department of General Surgery, Anhui Provincial Hospital, The First Affiliated Hospital of USTC, Division of Life Science and Medicine, University of Science and Technology of China, Hefei 230027, China; The Chinese Academy of Sciences Key Laboratory of Innate Immunity and Chronic Disease, School of Basic Medical Sciences, Division of Life Science and Medicine, University of Science and Technology of China, Hefei 230027, China; Department of General Surgery, Anhui Provincial Hospital, The First Affiliated Hospital of USTC, Division of Life Science and Medicine, University of Science and Technology of China, Hefei 230027, China; The Chinese Academy of Sciences Key Laboratory of Innate Immunity and Chronic Disease, School of Basic Medical Sciences, Division of Life Science and Medicine, University of Science and Technology of China, Hefei 230027, China; Insitute of Health and Medicine, Hefei Comprehensive National Science Center, Hefei 230601, China; Department of General Surgery, Anhui Provincial Hospital, The First Affiliated Hospital of USTC, Division of Life Science and Medicine, University of Science and Technology of China, Hefei 230027, China; The Chinese Academy of Sciences Key Laboratory of Innate Immunity and Chronic Disease, School of Basic Medical Sciences, Division of Life Science and Medicine, University of Science and Technology of China, Hefei 230027, China; Medical Research Institute, Guangdong Provincial People’s Hospital, Guangdong Academy of Medical Sciences, Southern Medical University, Guangzhou 510080, China; Department of General Surgery, Anhui Provincial Hospital, The First Affiliated Hospital of USTC, Division of Life Science and Medicine, University of Science and Technology of China, Hefei 230027, China; The Chinese Academy of Sciences Key Laboratory of Innate Immunity and Chronic Disease, School of Basic Medical Sciences, Division of Life Science and Medicine, University of Science and Technology of China, Hefei 230027, China; The Chinese Academy of Sciences Key Laboratory of Innate Immunity and Chronic Disease, School of Basic Medical Sciences, Division of Life Science and Medicine, University of Science and Technology of China, Hefei 230027, China; The Chinese Academy of Sciences Key Laboratory of Innate Immunity and Chronic Disease, School of Basic Medical Sciences, Division of Life Science and Medicine, University of Science and Technology of China, Hefei 230027, China; Medical Research Institute, Guangdong Provincial People’s Hospital, Guangdong Academy of Medical Sciences, Southern Medical University, Guangzhou 510080, China; The Chinese Academy of Sciences Key Laboratory of Innate Immunity and Chronic Disease, School of Basic Medical Sciences, Division of Life Science and Medicine, University of Science and Technology of China, Hefei 230027, China; Medical Research Institute, Guangdong Provincial People’s Hospital, Guangdong Academy of Medical Sciences, Southern Medical University, Guangzhou 510080, China; Department of General Surgery, Anhui Provincial Hospital, The First Affiliated Hospital of USTC, Division of Life Science and Medicine, University of Science and Technology of China, Hefei 230027, China; The Chinese Academy of Sciences Key Laboratory of Innate Immunity and Chronic Disease, School of Basic Medical Sciences, Division of Life Science and Medicine, University of Science and Technology of China, Hefei 230027, China; Department of General Surgery, Anhui Provincial Hospital, The First Affiliated Hospital of USTC, Division of Life Science and Medicine, University of Science and Technology of China, Hefei 230027, China; Department of General Surgery, Anhui Provincial Hospital, The First Affiliated Hospital of USTC, Division of Life Science and Medicine, University of Science and Technology of China, Hefei 230027, China; The Chinese Academy of Sciences Key Laboratory of Innate Immunity and Chronic Disease, School of Basic Medical Sciences, Division of Life Science and Medicine, University of Science and Technology of China, Hefei 230027, China; Insitute of Health and Medicine, Hefei Comprehensive National Science Center, Hefei 230601, China

**Keywords:** acetylation, lactylation, liver cancer, PDC, PDHX

## Abstract

Deactivation of the mitochondrial pyruvate dehydrogenase complex (PDC) is important for the metabolic switching of cancer cell from oxidative phosphorylation to aerobic glycolysis. Studies examining PDC activity regulation have mainly focused on the phosphorylation of pyruvate dehydrogenase (E1), leaving other post-translational modifications largely unexplored. Here, we demonstrate that the acetylation of Lys 488 of pyruvate dehydrogenase complex component X (PDHX) commonly occurs in hepatocellular carcinoma, disrupting PDC assembly and contributing to lactate-driven epigenetic control of gene expression. PDHX, an E3-binding protein in the PDC, is acetylated by the p300 at Lys 488, impeding the interaction between PDHX and dihydrolipoyl transacetylase (E2), thereby disrupting PDC assembly to inhibit its activation. PDC disruption results in the conversion of most glucose to lactate, contributing to the aerobic glycolysis and H3K56 lactylation-mediated gene expression, facilitating tumor progression. These findings highlight a previously unrecognized role of PDHX acetylation in regulating PDC assembly and activity, linking PDHX Lys 488 acetylation and histone lactylation during hepatocellular carcinoma progression and providing a potential biomarker and therapeutic target for further development.

## Introduction

Accumulating evidence demonstrates that cancer cells exhibit distinct metabolic features compared to their normal counterparts (Faubert et al., 2020; Pavlova et al., 2022), frequently relying on aerobic glycolysis to acquire energy, metabolites, and NADPH to maintain redox balance for their survival and proliferation. The altered metabolic function of cancer cells with increased utilization of aerobic glycolysis and higher lactate production is known as the Warburg effect (DeBerardinis and Chandel, 2020; Liberti and Locasale, 2016; Vander Heiden et al., 2009). Such altered cellular metabolism is mainly attributed to a decrease in the activity of enzymes participating in the tricarboxylic acid (TCA) cycle (Mullen and DeBerardinis, 2012), and in particular, pyruvate dehydrogenase complex (PDC), which links glycolysis to the TCA cycle to play a central role in glucose metabolism (Park et al., 2018). PDC is a molecular complex composed of pyruvate dehydrogenase (PDH), dihydrolipoamide dehydrogenase (DLD, E3), and a core made up of dihydrolipoyl transacetylase (DLAT, E2) and pyruvate dehydrogenase complex component X (PDHX) . Within PDC, PDH and DLD bind to DLAT and PDHX, respectively (Hiromasa et al., 2004; Patel and Korotchkina, 2001; Prajapati et al., 2019; Smolle et al., 2006). Since PDH catalyzes the rate-limiting step of pyruvate decarboxylation, thus determining the rate of PDC flux. Most studies examining the regulation of PDC activity have therefore focused on the phosphorylation of PDH by specific PDH kinases (PDKs), which inhibit PDC activity through phosphorylating PDH, and PDH phosphatases (PDPs) that function as PDC activators (Patel et al., 2014). For example, it has been shown that PDK1 is upregulated by Myc and hypoxia-inducible factor-1a (HIF-1a), inhibiting PDC and promoting Warburg effect in cancer cells (Kim et al., 2006; Papandreou et al., 2006). While the phosphorylation regulation of PDH has been well described, other mechanisms regulating the activity of the PDC potentially by altering complex assembly and function, remain largely unknown.

Lysine acetylation has been described as a general post-translational modification (PTM) of both histone and non-histone proteins (Narita et al., 2019; Shvedunova and Akhtar, 2022; Sun et al., 2022), with roles in regulating a wide variety of cellular processes, including metabolic reprogramming (Wang et al., 2010; Zhao et al., 2010). Our previous study revealed that the acetylation-dependent deactivation of SDHA (succinate dehydrogenase complex subunit A) alters the epigenetic regulation of gene expression, promoting tumorigenesis (Li et al., 2020). In addition to the regulating enzyme activity and/or stability, lysine acetylation has also been reported to regulate protein–protein interactions (Wang et al., 2016; Zhang et al., 2023). Interestingly, lysine acetylation is also involved in regulating PDC (An et al., 2023; Fan et al., 2014). Lys 321 acetylation of PDHA1 was shown to inhibit its activity by recruiting PDK1, and Lys 202 acetylation of PDP1 inhibits PDC activity by promoting the disassociation of its substrate PDHA1 (Fan et al., 2014). However, these acetylation-mediated effects on PDC activity are also attributed to the regulation of PDH phosphorylation, while any potential effects of acetylation on PDC assembly and activity by altering the interactions between PDC components remains unexplored.

Lactate, long recognized as a metabolic waste product of glycolysis, has recently been shown to contribute to tumor progression, including promoting tumor cell proliferation by providing energy during tumorigenic processes and promoting the evasion of immune surveillance through creating an acidic microenvironment (Certo et al., 2021; Hui et al., 2017; Kumagai et al., 2022). Apart from its important metabolic function, lactate was also recently described to generate a previously undescribed PTM, lysine lactylation (Kla), on core histones, and proven to epigenetically turn on M2-like gene expression in bacteria-challenged macrophages (Zhang et al., 2019). However, the effect of the novel Kla modification on gene expression in cancer cells remains unclear.

In this study, we demonstrate that the acetylation of PDHX Lys 488 by the p300 impedes the interaction between PDHX and DLAT, preventing the assembly of the PDC core and abolishing PDC activity. As a result, lactate production is enhanced in cancer cells, which contributes to the Warburg effect and H3K56 lactylation, which alters the transcriptional profile to promote tumor progression. These findings underline a previously unrecognized role of PDHX acetylation in regulating PDC assembly and activity, revealing a link between PDHX Lys 488 acetylation and histone lactylation during HCC progression. These findings nominate PDHX Lys 488 acetylation as an HCC biomarker and a potential therapeutic target for treatment development.

## Results

### PDHX acetylation at Lys 488 is upregulated in HCC and correlated with poor clinical prognosis

**Figure 1.**
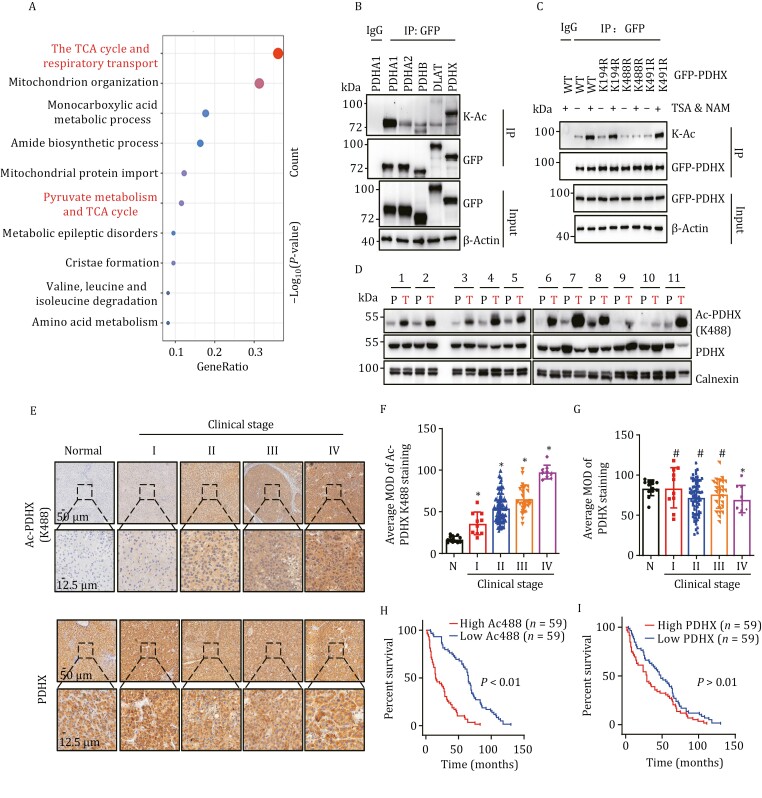
PDHX acetylation at Lys 488 is upregulated in HCC and correlated with poor clinical prognosis. (A) Pathway and process enrichment (top10) bubble plot derived from data of nano LC–MS/MS. (B) Immunoprecipitation (IP) assay was performed in HEK293T cells transfected with GFP-tagged PDHA1, PDHA2, PDHB, DLAT, or PDHX plasmids. Cells were treated with TSA (5 μmol/L, 16 h) and NAM (10 mmol/L, 8 h). (C) IP assay was performed in HEK293T cells transfected with GFP-PDHX-WT (WT), GFP-PDHX-K194R (K194R), GFP-PDHX-K488R (K488R) or GFP-PDHX-K491R (K491R) plasmids. Cells were treated with or without TSA (5 μmol/L, 16 h) and NAM (10 mmol/L, 8 h). (D) Immunoblotting analysis of PDHX Lys 488 acetylation and PDHX expression in the paired tumor-adjacent noncancerous liver tissues (P) and human HCC tissues (T) (*n* = 11). Calnexin served as a loading control. (E) Representative IHC images of PDHX and PDHX Lys 488 acetylation staining in normal liver tissue (normal) and HCC specimens of different clinical stages (I–IV); scale bars, 50 μm. Insets: 4-fold magnification; scale bars, 12.5 μm. (F and G) Statistical quantification of MOD values for PDHX Lys 488 acetylation (F) and PDHX staining (G) in IHC assays between normal liver tissues and HCC specimens at clinical stages I–IV (healthy donors, *n* = 13; patients with HCC, stage I (*n* = 10), II (*n* = 73), III (*n* = 26) and IV (*n* = 9)). Data presented as mean ± SEM Statistical significance was determined by two-tailed unpaired Student’s *t*-test., *P < 0.05, compared with normal (N) group. #, not significant. (H and I) Kaplan–Meier curves with univariate analyses of patients with low versus high expression of PDHX Lys 488 acetylation (H) (high PDHX Lys 488 acetylation, *n* = 59 patients; low PDHX Lys 488 acetylation, *n* = 59 patients) or PDHX (I) (high PDHX, *n* = 59 patients; low PDHX, *n* = 59 patients). High and low expression is defined by the median value calculated from IHC staining data of HCC patient samples. Statistical significance was determined by log-rank test.

Protein acetylation is linked to cancer metabolism (Wang et al., 2010; Zhao et al., 2010) and the shift from oxidative phosphorylation to glycolysis is the most prominent metabolic change associated with cancer progression. However, little is known regarding the role of acetylation in this metabolic shift. To identify proteins that are acetylated in the mitochondria and might take part in metabolic shift, nanoscale liquid chromatography separation and high-resolution mass spectrometry (nano LC-MS/MS) analysis was performed on enriched acetylated mitochondrial peptides isolated from P493 cells. Nano LC-MS/MS identified 454 lysine-acetylated peptides, matching 150 distinct proteins. To determine the function of the 150 proteins, we performed Gene Ontology term enrichment analysis, revealing their participation in the TCA cycle and pyruvate metabolism, which were among the top 10 enriched pathways (Fig. 1A). Notably, most PDC components exhibited lysine-acetylation modification (Fig. S1A). To determine if these proteins are acetylated, we created GFP fusion constructs encoding each of the PDC subunits and transfected them into HEK293T cells. Cells expressing the fusion constructs were treated with nicotinamide (NAM) and trichostatin A (TSA) to inhibit endogenous deacetylases, and protein lysates from cells expressing each fusion construct were used in an immunoprecipitation (IP) assay to pull down GFP (Fig. 1B). IP reactions were blotted using an antibody recognizing K-Ac, revealing that each of the exogenous GFP-tagged PDC subunits were acetylated. Among the subunits, PDHA1 and PDHX had particularly high levels of the lysine-acetylation PTM (Figs. 1B and S1B). Since PDHA1 acetylation was previously reported (Fan et al., 2014), we focused on exploring the role of PDHX acetylation in cancer cells. Reverse IP using anti-acetylated-lysine antibody to pulldown lysine acetylated proteins from HEK293T lysates expressing Flag-PDHX further confirmed that PDHX is acetylated, and PDHX acetylation was enhanced after NAM and TSA treatment (Fig. S1C).

Mass spectrometry analysis revealed that Lys 194, Lys 488, and Lys 491 of PDHX were potential acetylation sites. To validate the potential lysine residue(s) on PDHX, each residue was individually mutated in the GFP–PDHX fusion construct to the nonacetylatable arginine (R) residue and the resulting constructs were transfected into HEK293T cells. GFP IP followed by blotting for K-Ac revealed that acetylation was largely abolished in the K488R mutant, but not in the K194R and K491R mutants (Fig. 1C), suggesting Lys 488 is the critical acetylation site of PDHX. Similar results were also observed in HCC cells (Fig. S1D–F). In order to detect Lys 488-acetylated PDHX, we created an antibody to recognize the TRFLK(Ac)SFKA epitope, which was verified using a dot blot assay (Fig. S1G and S1H). The antibody could recognize wild type PDHX, but not K488R and K488Q mutant, in HepG2 cells overexpressing these constructs (Fig. S1I). Together, these data indicated that PDHX is acetylated at Lys 488.

HCC cell metabolism exhibits high utilization of aerobic glycolysis compared with non-cancerous cells (Feng et al., 2020), and altered HCC metabolism may be due, at least in part to the inhibition of PDC. To investigate the potential pathological significance of PDHX Lys 488 acetylation in HCC, we examined the expression levels of PDHX Lys 488 acetylation and PDHX in 11 paired clinical samples of human HCC lesions and the adjacent non-cancerous tissue. The results revealed that the level of PDHX Lys 488 acetylation was significantly increased in HCC lesions compared to adjacent non-cancerous tissue, while level of total PDHX remained stable (Fig. 1D). Subsequently, the IHC assays revealed that PDHX Lys 488 acetylation was generally negative in normal human liver tissues, but was positively correlated with higher clinical stages of HCC, with total PDHX maintained high expression at different stages (Fig. 1E). Quantification of the staining intensity of normal and HCC sections further demonstrated PDHX Lys 488 acetylation, but not total PDHX expression, to be positively correlated with HCC progression (Fig. 1F and 1G). Finally, the Kaplan–Meier test indicated that PDHX Lys 488 acetylation, but not total PDHX expression was significantly associated with patient survival time (Fig. 1H and 1I), suggesting that the level of PDHX Lys 488 acetylation could potentially serve as a prognostic biomarker for HCC (all statistically significant; Tables S1–3). Collectively, our cellular and clinical data revealed that PDHX Lys 488 acetylation is upregulated in HCC, and is highly associated with liver cancer progression.

### PDHX Lys 488 is acetylated by p300 in the cytoplasm

**Figure 2.**
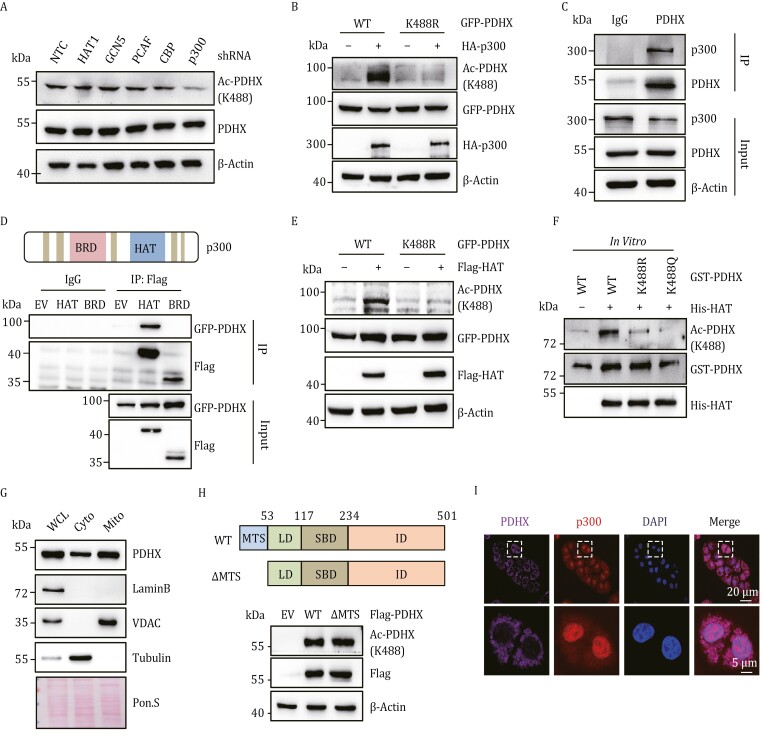
PDHX Lys 488 is acetylated by p300 in the cytoplasm. (A) Immunoblotting analysis of PDHX Lys 488 acetylation and PDHX protein levels in HepG2 cells expressing NTC or shRNA targeting p300, CBP, PCAF, HAT1 or GCN5. (B) HEK293T cells were transfected with GFP-PDHX-WT or GFP-PDHX-K488R, followed by further transfection with EV or HA-p300. Immunoblotting analysis of PDHX Lys 488 acetylation, HA and GFP levels. (C) Endogenous IP was performed using anti-PDHX antibody or IgG in HepG2 cells. Immunoblotting analysis of PDHX and p300 levels. (D) Co-IP assay in HEK293T cells co-transfected with GFP-PDHX and Flag-tagged BRD or HAT domain plasmids. (E) Immunoblotting analysis of PDHX Lys 488 acetylation, Flag and GFP levels in HEK293T cells co-transfected with GFP-PDHX-WT or GFP-PDHX-K488R and Flag-HAT. (F) His-HAT and GST-tagged PDHX WT, K488R and K488Q were purified from *E. coli*, and then *in vitro* acetylation analyses were performed by mixing purified His-HAT with the purified GST-PDHX WT, K488R and K488Q proteins in the presence of Ac-CoA. (G) Immunoblotting analysis of PDHX, LaminB1, VDAC and Tubulin in whole-cell lysates (WCL), cytoplasm lysates (Cyto) and mitochondria lysates (Mito) from HepG2 cells. Ponceau staining served as the loading control. (H) A schematic diagram of the Flag-PDHX-WT and Flag-PDHX-ΔMTS (upper panel). Immunoblotting analysis of PDHX Lys 488 acetylation and Flag in HepG2 cells infected with viruses expressing Flag-PDHX-WT or Flag-PDHX-ΔMTS (lower panel). (I) Representative images of immunofluorescence staining for p300 and PDHX in PLC cells. The nucleus was stained with DAPI. Scale bars, 20 μm. Insets: fourfold magnification; scale bars, 5 μm.

To identify which acetyltransferase(s) are responsible for PDHX Lys 488 acetylation, we performed an shRNA screen examining HAT1, GCN5, PCAF, CBP and p300. Knockdown of p300, but not the other HAT proteins, significantly decreased PDHX Lys 488 acetylation (Figs. 2A and S2A), suggesting that p300 might be involved in regulating PDHX Lys 488 acetylation. Treating HepG2 cells with the p300 acetyltransferase inhibitors C646 and A-485 resulted in decreased PDHX Lys 488 acetylation, while treatment with the p300 bromodomain inhibitor UMB298 had no effect (Fig. S2B). Moreover, PDHX Lys 488 acetylation was enhanced by overexpressing p300, but the acetylation level of PDHX K488R mutant was not affected by p300 (Fig. 2B). Additionally, we and others found that p300 was highly expressed in liver cancer (Fig. S2C) (Li et al, 2011; Yokomizo et al, 2011). Altogether, these data confirmed that p300 promotes PDHX acetylation at Lys 488.

IP PDHX and blotting for p300 revealed that endogenous p300 interacts with PDHX in HepG2 cells (Fig. 2C). To determine if the p300 HAT domain or BRD domain was required for the interaction, Flag-p300-BRD and Flag-p300-HAT constructs were overexpressed in HEK293T cells, Co-IP revealed that PDHX interacts with the p300 HAT and not the p300 BRD (Fig. 2D). Furthermore, GST pulldown experiments showed the direct interaction between PDHX and p300 HAT (Fig. S2D). These data indicated that PDHX might be directly acetylated by p300 HAT domain, the minimally required domain for p300 acetyltransferase function (Liu et al., 2008; Thompson et al., 2004). We verified this observation by overexpressing the p300 HAT domain and examining levels of PDHX Lys 488 acetylation. Overexpression of the p300 HAT domain was able to increase PDHX acetylation at Lys 488 but did not alter acetylation levels of PDHX K488R mutant in cells (Fig. 2E). We further confirmed this observation by mixing purified p300 HAT and GST PDHX proteins *in vitro*, obtaining the same result (Fig. 2F). Together, these data show that the HAT domain of p300 directly interacts with PDHX to acetylate it at Lys 488.

Within cells, p300 is mainly located in the nucleus and cytoplasm, while PDHX localized in mitochondria, raising the question of where the PDHX–p300 interaction occurs to acetylate PDHX. Since PDHX protein is translated in the cytoplasm before being transported into the mitochondria, we considered that PDHX might be acetylated in the cytoplasm by p300 before translocation. To validate this hypothesis, we performed cell fractionation assays to detect the levels of PDHX protein in the cytoplasm and mitochondria. As expected, PDHX protein is present at high levels in both the cytoplasm and mitochondria (Fig. 2G). We next generated MTS (mitochondria translocation sequence)-deleted PDHX (ΔMTS-PDHX), which cannot be transported into the mitochondria (Fig. S2E). Notably, ΔMTS-PDHX was still acetylated at comparable levels to wild-type PDHX (Fig. 2H). Furthermore, immunofluorescence staining experiment also indicated that p300 and PDHX are co-localized in the cytosol (Fig. 2I). These results confirmed that the acetylation of PDHX occurs in the cytoplasm. Indeed, some studies have shown that acetylation can affect the localization of proteins in cells (Gao et al, 2020; Li et al, 2022). So, we detected the mitochondrial localization of PDHX-WT, -K488R, and -K488Q in HepG2 cells. Our results showed that there were no detectable differences in protein subcellular localization among PDHX-WT, -K488R, and -K488Q (Fig. S2F). Collectively, our results demonstrated that p300 plays a crucial role in facilitating the acetylation of PDHX at Lys 488 in the cytoplasm.

### PDHX Lys 488 acetylation-induced dissociation between PDHX and DLAT inhibits PDC assembly and activity

**Figure 3.**
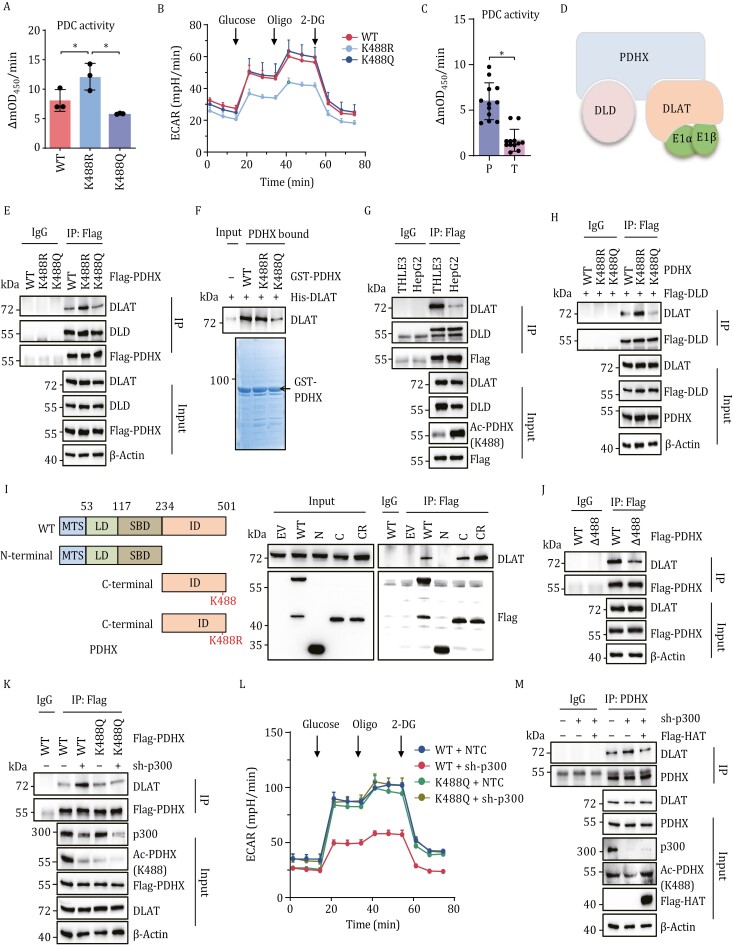
PDHX Lys 488 acetylation-induced dissociation between PDHX and DLAT inhibits PDC assembly and activity. (A) PDC activity measurements in HepG2 cells expressing with PDHX-WT, PDHX-K488R or PDHX-K488Q. Data are presented as the mean ± SD of three independent experiments (*n* = 3). *, *P* < 0.05, compared with K488R group. (B) The ECAR was measured by successive injections of Glucose, oligomycin (Oligo) and 2-DG in HepG2 cell expressing with PDHX-WT, PDHX-K488R or PDHX-K488Q. Data are presented as the mean ± SD of three independent experiments (*n* = 3). (C) PDC activity measurements in the paired tumor-adjacent noncancerous liver tissues (P) and human HCC tissues (T) (mean ± SEM of *n* = 12 biologically independent experiments). *P < 0.05, compared with HCC tissues (T) group. (D) A schematic diagram of the PDC. (E) IP was performed using anti-Flag antibody or IgG in HepG2 cells expressing Flag-PDHX-WT, Flag-PDHX-K488R or Flag-PDHX-K488Q and Immunoblotting analysis of Flag, DLD, and DLAT. (F) Different GST-PDHX mutants were purified from *E. coli*, and then pull-down assay was performed. (G) IP was performed using anti-Flag antibody or IgG in HepG2 or THLE3 cells overexpressing Flag-PDHX-WT. Immunoblotting analysis of DLAT, DLD, PDHX Lys 488 acetylation and Flag levels. (H) HepG2 cells overexpressing Flag-DLD were further infected with viruses expressing PDHX-WT, PDHX-K488R or PDHX-K488Q. IP was performed using anti-Flag antibody or IgG. (I) A schematic diagram of the PDHX-WT, N-terminal, C-terminal and C-terminal with K488R mutation (left panel). IP was performed using anti-Flag antibody or IgG in HEK293T cells transfected with Flag-tagged PDHX-WT, N-terminal, C-terminal and C-terminal with K488R mutation (right panel). (J) IP was performed using anti-Flag antibody or IgG in HepG2 cells overexpressing Flag-PDHX-WT or Flag-PDHX-Δ488 (delete Lys 488). (K) HepG2 cells overexpressing Flag-PDHX-WT or Flag-PDHX-K488Q were further infected with viruses expressing NTC or shp300. IP was performed using anti-Flag antibody or IgG. (L) HepG2 cells overexpressing Flag-PDHX-WT or Flag-PDHX-K488Q were further infected with viruses expressing NTC or shp300. The ECAR was measured by successive injections of Glucose, oligomycin (Oligo) and 2-DG. (M) HepG2 cells stably expressing NTC or p300 shRNA were further infected with viruses for expression of Flag-EV or Flag-p300-HAT. IP was performed using PDHX antibody or IgG.

Lysine acetylation has been shown to affect the activity of a number of different enzymes (Han et al., 2023; Lv et al., 2011). Considering that PDHX is the core component of the PDC, we next investigated whether PDHX Lys 488 acetylation could modulate PDC activity. To examine whether acetylation alters PDC function, endogenous PDHX was depleted in HepG2 cells and either PDHX-WT, non-acetylatable PDHX K488R, or acetyl-mimetic PDHX K488Q were overexpressed. Our data revealed that cells expressing the PDHX K488R mutant exhibited relatively high PDC activity, while cells expressing the K488Q mutant possessed low PDC activity (Fig. 3A), suggesting that PDHX Lys 488 acetylation negatively affects PDC activity. Given the role of PDC in aerobic glycolysis, we further measured the extracellular acidification rate (ECAR) in these cells, revealing a reduction in both the basal and maximal ECAR in HepG2 cells expressing the K488R mutant (Figs. 3B and S3A). PDC activity levels were also shown to be significantly lower in patients with clinical liver cancer compared to healthy subjects (Fig. 3C), an observation that could be attributed to high levels of PDHX Lys 488 acetylation in patients (Fig. 1D–F). Collectively, these data suggested that PDHX Lys 488 acetylation inhibits PDC activity and contributes to metabolic reprogramming of cancer cells.

PDC is composed of PDH (E1), DLAT, DLD, and PDHX (Fig. 3D). The acetylation of PDHX, an E3-binding protein (E3BP) lacking catalytic activity, could not directly be responsible for reduced PDC activity. In addition, lysine acetylation has also been reported to regulate interactions between proteins (Huang et al., 2022; Wang et al., 2016). Therefore, we tested whether PDHX Lys 488 acetylation might inhibit PDC activity through disrupting the interaction of PDHX with DLAT or DLD. Flag-tagged PDHX WT, PDHX K488R, and PDHX K488Q were overexpressed in HepG2 cells and immunoprecipitated using a Flag antibody, followed by blotting for DLAT and DLD. We found that PDHX Lys 488 acetylation blocked the interaction between PDHX and DLAT, but not the interaction between PDHX and DLD (Fig. 3E). Further GST pull-down experiments using purified GST-tagged PDHX WT, PDHX K488R and PDHX K488Q demonstrated that PDHX interacts directly with DLAT, and that the interaction is impaired by PDHX Lys 488 acetylation (Fig. 3F). Interestingly, since PDHX is highly acetylated in HepG2 cells, the PDHX–DLAT interaction was weaker in this cell line compared to that in normal hepatocytes (Fig. 3G). Previous reports and our result revealed that the interaction between DLAT and DLD is PDHX-dependent (Fig. S3B). We therefore asked if PDHX Lys 488 acetylation inhibits the interaction between DLAT and DLD, to inhibit PDC assembly. To test this, Flag-DLD was overexpressed in combination with PDHX WT, PDHX K488R, or PDHX K488Q, and cell lysates were immunoprecipitated for Flag. Notably, the acetyl-mimetic PDHX K488Q inhibited the interaction between DLAT and DLD (Fig. 3H). Taken together, these results show that PDHX Lys 488 acetylation results in diminished PDC assembly in cancer cells.

To identify the domains required for the interaction between PDHX and DLAT, the full-length PDHX was divided into the N-terminal domain, the C-terminal domain, and the C-terminal domain with K488R mutation (Fig. 3I, left). Co-IP experiments demonstrated that the C-terminal domain of PDHX, which contains Lys488 site, is responsible for the interaction between PDHX and DLAT, and that the interaction is enhanced when Lys 488 is deacetylated (Fig. 3I, right). We further generated a Lys 488-deletion PDHX (Δ488-PDHX) and examined the interaction with DLAT. The interaction between PDHX and DLAT could be suppressed when Lys 488 was deleted, confirming the participation of the Lys 488 residue in forming the interaction with DLAT (Fig. 3J).

Next, we investigated whether p300, which plays a role in promoting the acetylation of PDHX at Lys 488, influences the interaction between PDHX and DLAT and PDC activity. While knockdown of p300 could enhance the interaction between PDHX WT and DLAT, the acetyl-mimetic K488Q mutant abolished the facilitatory effect (Fig. 3K). Similar results were observed by inhibiting the p300 HAT using C646 (Fig. S3C). To examine PDC activity, the ECAR was measured. We observed a significant reduction in both basal and maximal ECAR in HepG2 cells expressing PDHX WT with p300 depletion, compared to other conditions (Figs. 3L and S3D). Notably, restoring the expression of the p300 HAT domain after knockdown of endogenous p300 reduced the levels of the interaction between PDHX and DLAT to a similar level in HepG2 cells without p300 knockdown (Fig. 3M). Collectively, these data show that p300 plays an important role in regulating PDC assembly and its activity.

### PDHX Lys 488 acetylation promotes H3K56 lactylation and gene expression

**Figure 4.**
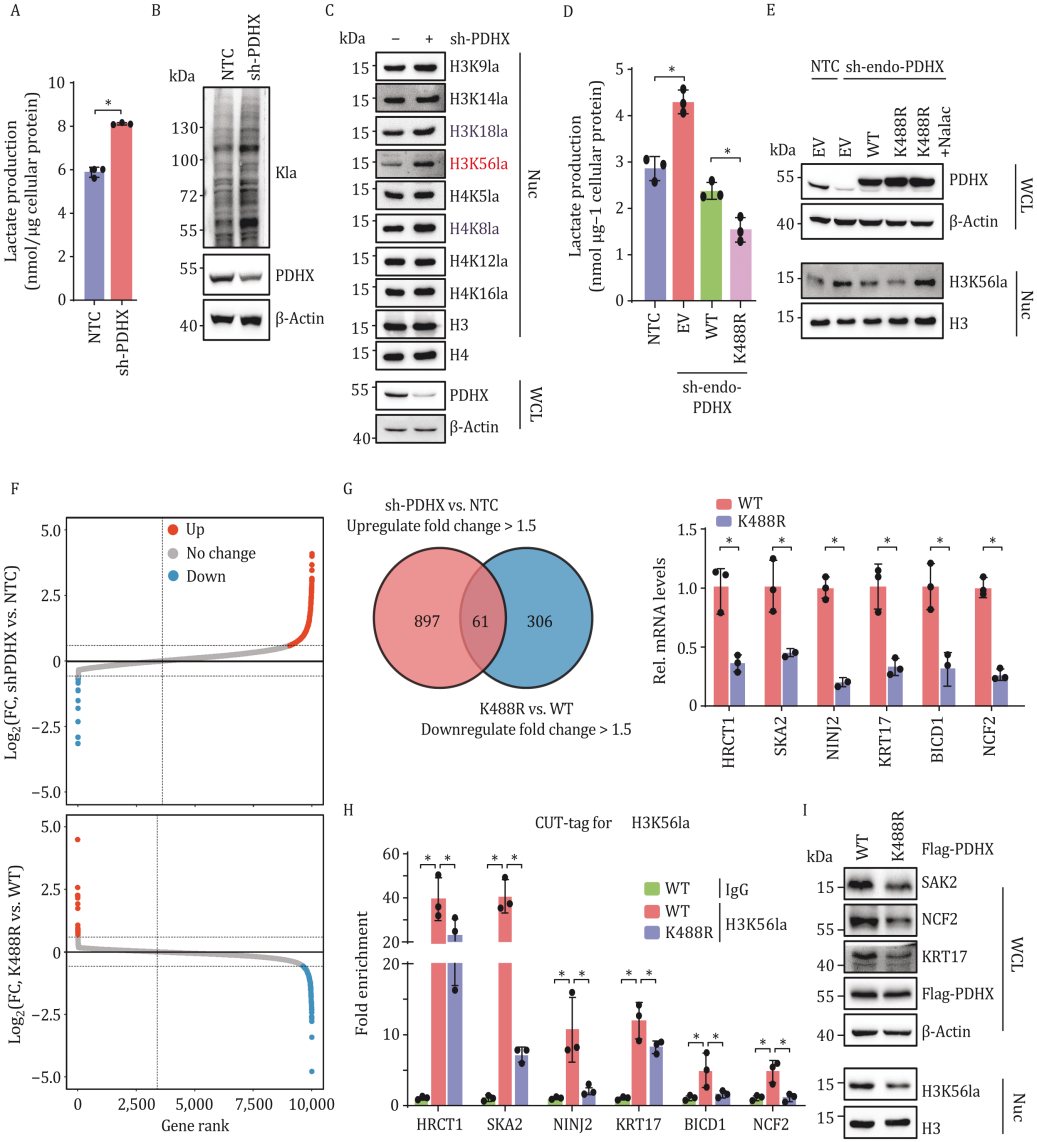
PDHX Lys 488 acetylation promotes H3K56 lactylation and gene expression. (A) HepG2 cells were transfected with NTC or shRNA targeting PDHX, followed by measurement of lactate production in culture medium. Data are presented as the mean ± SD of three independent experiments (*n* = 3). *P < 0.05, compared with NTC group. (B) Immunoblotting analysis of pan lactylation in HepG2 cells transfected with NTC or shRNA for PDHX. (C) Immunoblotting analysis of histone lactylation in HepG2 cells transfected with NTC or shRNA for PDHX. (D) HepG2 cells with endogenous PDHX knockdown were subsequently infected with viruses of EV, PDHX-WT or PDHX-K488R followed by measurement of lactate production in culture medium. Data are presented as the mean ± SD of three independent experiments (*n* = 3). *P < 0.05, between the indicated groups. (E) HepG2 cells with endogenous PDHX knockdown were subsequently infected with viruses of EV, PDHX-WT, PDHX-K488R or PDHX-K488R with Nalac treatment. Immunoblotting analysis of H3K56 lactylation. (F) RNA-seq analysis of HepG2 cells expressing NTC or shPDHX. Candidate genes were plotted based on mean log_2_-fold change of RNA counts compared to control NTC group. Blue dots indicate downregulated genes whereas red dots indicate upregulated genes by shPDHX (fold-change > 1.5) (upper panel). RNA-seq analysis of HepG2 cells with endogenous PDHX knockdown further expressing PDHX-WT or PDHX-K488R. Candidate genes were plotted based on mean log_2_ fold-change of RNA counts compared to control WT group. Blue dots indicate downregulated genes whereas red dots indicate upregulated genes by PDHX K488R (fold-change > 1.5) (lower panel). (G) Venn diagram of the RNA-seq data showing the genes regulated by both PDHX and PDHX Lys 488 acetylation (left panel). The mRNA levels of indicated oncogenes were determined by quantitative real-time PCR (qRT–PCR) in the HepG2 cells expressing PDHX-WT or K488R (right panel). Data are presented as the mean ± SD of three independent experiments (*n* = 3). *P < 0.05, compared with WT group. (H) CUT&tag assay analysis of the occupancy of H3K56la on the indicated gene promoters in the HepG2 cells expressing PDHX-WT or K488R. Data are presented as the mean ± SD of three independent experiments (*n* = 3). *P < 0.05, between the indicated groups. (I) The indicated proteins were assessed by immunoblotting in the HepG2 cells used in (H).

PDC deactivation is involved in metabolic reprogramming and lactate overproduction in cancer cells. To determine the role of PDHX in PDC activation and its effects on lactate production, we measured the lactate content in HepG2 cells after PDHX depletion. As expected, knocking down PDHX results in elevated levels of lactate in HepG2 cells (Fig. 4A). Lactate was recently shown to be covalently linked to proteins, forming a novel type of PTM with regulatory function, with histone lactylation representing a form of epigenetic modification critical for many biological processes (Rho et al., 2023; Sun et al., 2023; Zhang et al., 2019). Therefore, we tested whether the accumulation of lactate induced by PDHX-depletion promotes protein lactylation in cancer cells. Overall, the global protein lactylation in HepG2 cells was increased by depletion of PDHX (Fig. 4B). We then specifically explored whether lactate accumulation downstream of PDHX deficiency altered levels of histone lactylation. Notably, among the histones lactylation modifications that we tested, H3K56la was dramatically increased after suppression of PDHX in cancer cells (Fig. 4C). These results suggested that PDC deactivation downstream of PDHX inactivation increase cellular lactate production and results in the lactylation of H3K56.

Next, we investigated whether PDHX Lys 488 acetylation regulates cellular lactate production and H3K56la. PDHX depletion resulted in increased lactate production in HepG2 cells, which was rescued by overexpressing PDHX WT. Overexpressing the non-acetylatable PDHX K488R reduced lactate production to a significantly lower level than WT HepG2 cells (Fig. 4D). Next, we examined levels of H3K56la after PDHX inactivation. After depleting endogenous PDHX in HepG2 cells, the overexpression of WT and to a greater extent K488R PDHX were capable of reducing H3K56la levels. However, treating HepG2 cells overexpressing PDHX K488R with exogenous Nalac resulted in increased H3K56la levels, suggesting that disrupting the tight control of cellular lactate levels maintained by PDC results in abnormal levels of the lactylation histone PTM (Fig. 4E).

We next examined how altering the function of these enzymatic pathways can affect the global gene expression profile of cancer cells. To examine how increased H3K56la levels downstream of PDHX acetylation affect gene expression, we performed twice RNA sequencing analyses (RNA-seq) in HepG2 cells. We compared the transcriptomes of cells expressing NTC or shPDHX by RNA-seq, 958 genes expressed at significantly greater levels after PDHX depletion (Fig. 4F, upper panel). To further determine genes that are specifically regulated by PDHX Lys 488 acetylation, we performed RNA-seq analysis in PDHX depleted cells overexpressing either PDHX WT or PDHX K488R, uncovering 367 genes that are suppressed by PDHX K488R mutation (Fig. 4F, lower panel). After combining the two RNA-seq analyses, 61 overlapping genes were identified, representing genes that are regulated by both PDHX and PDHX Lys 488 acetylation (Fig. 4G, left panel). qPCR analysis confirmed that PDHX K488R decreased the expression of several oncogenes [including *SKA2* (Xie and Bu, 2019), *KRT17* (Depianto et al., 2010), and *HRCT1* (Hou et al., 2023), etc.] that were identified among the overlapping 61 genes (Fig. 4G, right panel). Cleavage Under Target & Tagmentation (CUT&tag) assays further demonstrated that these oncogenes contained the H3K56la modification in their promoters, and that the H3K56la levels were reduced in cells expressing PDHX K488R compared to cells expressing PDHX WT (Fig. 4H). Finally, PDHX K488R was also shown to inhibit the expression of these oncogenes at protein level (Fig. 4I). Collectively, these results show that PDHX Lys 488 acetylation drives PDC deactivation, resulting in lactate overproduction and H3K56la, and consequently drives oncogene expression.

### PDHX Lys 488 acetylation contributes to the progression of glycolytic tumor

**Figure 5.**
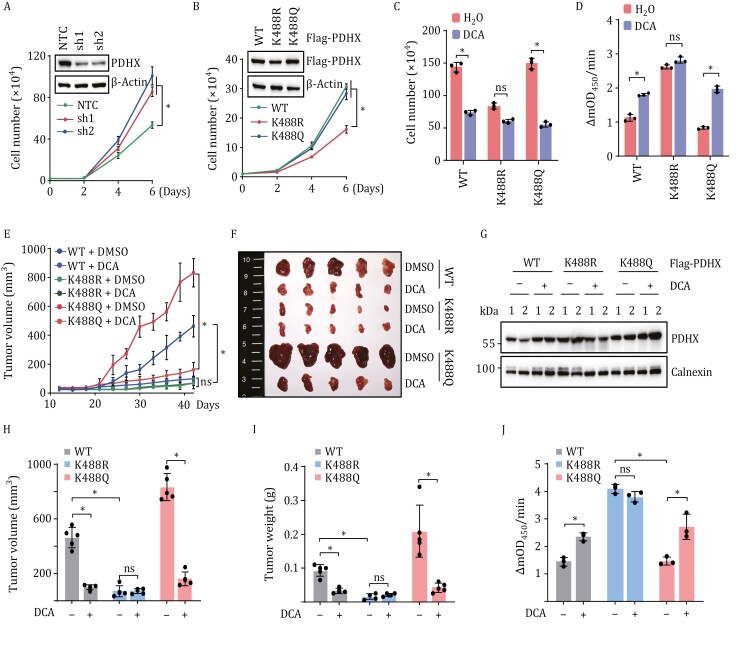
PDHX Lys 488 acetylation contributes to the progression of glycolytic tumor. (A) Relative growth curve of HepG2 cells with or without PDHX knockdown. Data are presented as the mean ± SD of three independent experiments (*n* = 3). *P < 0.05, compared with NTC group. Knockdown efficiency in HepG2 cells was verified by immunoblotting. (B) Relative growth curve of HepG2 cells with endogenous PDHX knockdown and further expressing PDHX-WT, PDHX-K488R or PDHX-K488Q. Data are presented as the mean ± SD of three independent experiments (*n* = 3). Expression efficiency in HepG2 cells was verified by immunoblotting. *P < 0.05, compared with K488R group. (C) HepG2 cells overexpressing PDHX-WT, PDHX-K488R or PDHX-K488Q were further treated with DCA for 48 h, followed by measurement of cell numbers. Data are presented as the mean ± SD of three independent experiments (*n* = 3). *P < 0.05, compared with control (H_2_O) group. ns, not significant. (D) Measurement of PDC activity in HepG2 cells used in (C), Data are presented as the mean ± SD of three independent experiments (*n* = 3). *P < 0.05, compared with control (H_2_O) group. ns, not significant. (E, F) HepG2 cells (5 × 10^6^) used in (B) were injected subcutaneously into BALB/c nude mice (*n* = 5, each group). Mice were treated with DCA (156 mg/kg body weigh) or PBS every three days starting from 9 days after inoculation. Tumor growth curves were measured starting from 12 days after inoculation. Data are presented as the mean ± SD of five independent experiments (*n* = 5). *P < 0.05, between the indicated groups. ns, not significant. (G) Immunoblotting analysis of Flag in the extracted tumors in (F), Calnexin was used as the loading control. (H) The tumor volume of the extracted tumors in (F) was measured. Data are presented as mean ± SD (*n* = 5, each group). Group differences are analyzed by the two-tailed Student’s *t*-test. *P < 0.05, between the indicated groups. ns, not significant. (I) The tumor weight of the extracted tumors in (F) was measured. Data are presented as mean ± SD (*n* = 5, each group). Group differences are analyzed by the two-tailed Student’s *t*-test. *P < 0.05, between the indicated groups. ns, not significant. (J) The PDC activity of the extracted tumors in (F) was measured. Data are presented as mean ± SD (*n* = 3, each group). Group differences are analyzed by the two-tailed Student’s *t*-test. *P < 0.05, between the indicated groups. ns, not significant.

PDC inactivation contributes to tumor progression in multiple cancer types (Eastlack et al., 2018; Ma et al., 2014; Nie et al., 2020). Thus, it is of interest to investigate the effect of PDHX Lys 488 acetylation, which also results in PDC deactivation, on tumor-cell proliferation. Knocking down PDHX was sufficient to promote the proliferation of HepG2 cells (Fig. 5A). To test whether PDHX Lys 488 acetylation effects cell proliferation, we stablished a stable PDHX depletion cell line with constitutive expression of an shRNA targeting PDHX and then overexpressed shRNA resistant PDHX WT, or the K488R and K488Q mutants. Intriguingly, K488R overexpression resulted in decreased cell proliferation compared with cells expressing PDHX WT or K488Q mutant in HepG2 cells (Fig. 5B). K488Q expressing cells proliferated at similar rates with cells expressing PDHX WT which might be explained by our observation that WT PDHX is highly acetylated in cancer cells.

The PDK inhibitor dichloroacetate (DCA) promotes PDC activity and flux (Whitehouse and Randle, 1973), and is used to target glycolysis-dependent tumors (Stacpoole, 2017). Since PDHX Lys 488 acetylation rewired glucose metabolism to glycolysis in cancer cells, we examined whether PDHX Lys 488 acetylation affects the therapeutic effect of DCA. When cells expressing PDHX WT or PDHX K488Q were treated with DCA, proliferation was significantly inhibited. In contrast, the proliferation of cells expressing PDHX K488R was hardly affected by DCA, indicating that cancer cells with high level of PDHX Lys 488 acetylation are the target of DCA drugs (Fig. 5C). PDC activity was increased in cells expressing PDHX WT or K488Q treated with DCA, but not in cells expressing PDHX K488R (Fig. 5D).

Consistent with the cell-growth data, mouse xenograft experiments using cells expressing PDHX WT, K488R or K488Q had similar results: DCA treatment inhibited the growth of tumors expressing PDHX WT and K488Q, but had no effect on those expressing PDHX K488R, and tumors composed of K488R expressing cells grew more slowly than those expressing the other PDHX variants (Fig. 5E–I). PDC activity, which was highest in cells expressing PDHX K488R, was not increased in cells expressing K488R but was increased in cells expressing PDHX WT or PDHX K488Q after DCA treatment (Fig. 5J). Taken together, these data demonstrated that PDHX Lys 488 acetylation is an important determinant of liver cancer proliferation, and that DCA treatment specifically target the growth of glycolysis-dependent tumors through enhancing PDC activity. This indicates that tumors with high levels of PDHX Lys 488 acetylation are suitable candidates for DCA treatment.

## Discussion

The Warburg effect commonly occurs in most cancer subtypes and is essential for glycolytic-tumor development (Liberti and Locasale, 2016; Vander Heiden et al., 2009). Despite being discovered over 100 years ago, the underling mechanisms promoting the Warburg effect have largely gone unidentified, especially under normoxia. Given the intricate linkage of cellular metabolism with PDC function (Park et al., 2018), we examined the mechanisms underlying PDC inhibition in cancer cells. These investigations determined that PDHX, a key protein that bridges the PDC complex, functions to regulate PDC assembly, thereby strongly effecting PDC activity to impact tumor progression. Specifically, we found that the Lys 488 acetylation on PDHX disrupts the interaction between PDHX and DLAT, inhibiting the assembly of the PDC core and preventing complex formation. Inhibition of the PDC complex activity promotes the switching of glucose metabolism to aerobic glycolysis, raising intracellular levels of lactate to promote H3K56la, an epigenetic regulator that promotes oncogene expression.

Previous studies examining the function of PDC focused on the regulation of PDHA(E1α) by kinases and phosphatases (Cai et al., 2020; Fan et al., 2014; Nie et al., 2020), leading to the use of PDK inhibitor DCA to promote PDC activity therapeutically in cancer patients (Haugrud et al., 2014; Shen et al., 2015). In this study, we show that the activity of PDC is also inhibited by the acetylation of PDHX, controlling complex assembly and activity. Our results extend that PDC activity may be regulated by diverse ways, providing a conceptual basis for new HCC biomarkers and potentially the development of new treatment strategies. In addition, through *in vitro* and *in vivo* experiments, we found that DCA can specifically inhibit glycolysis-dependent tumor growth, and tumors with high levels of PDHX Lys 488 acetylation are the suitable target of DCA drugs (Fig. 5C–H). In conclusion, our data suggest that PDHX acetylation plays important role in regulating PDC activity through a PDHA1 phosphorylation-independent manner. These findings provide new insights into the regulation of PDC enzyme activity and will lead to the improved identification of patients who should be treated with DCA.

PTMs are global regulators of cellular behavior, controlling the activities and functionality of many cellular components (Sun et al., 2022). Lysine acetylation has been found to play key roles in cellular metabolism (Lei et al., 2020; Zhao et al., 2013). Previous studies had focused on how PTMs modulate the stability and activity of enzymatic processes in metabolism. Our results show PTMs can also be used to regulate the assembly of macromolecular complexes to control their activity levels. These findings shed light on the importance of PTMs in the process of big complex assembly, and may serve to encourage further studies to discover innovative mechanisms regarding enzyme activity regulation. Another novel PTM, lysine lactylation has recently been discovered on both histone and non-histone proteins, strongly affecting on the behavior of diseases, including cancer (Chen et al., 2024; Sun et al., 2022; Yu et al., 2021). Considering metabolic tendency of cancer cells to produce high levels of lactate, we hypothesized that Lys lactylation might provide a link between cancer cell metabolism, tumorigenicity, and the response of patients to treatments. We verified this hypothesis by showing that the acetylation of PDHX, resulting in decreased PDC activity, raises cellular levels of lactate to promote H3K56la. We found that H3K56la was present within the promoters of oncogenes that are stimulated by the acetylation of PDHX, thereby providing new insights into understanding the contributions of glycolysis to tumor progression.

**Figure 6.**
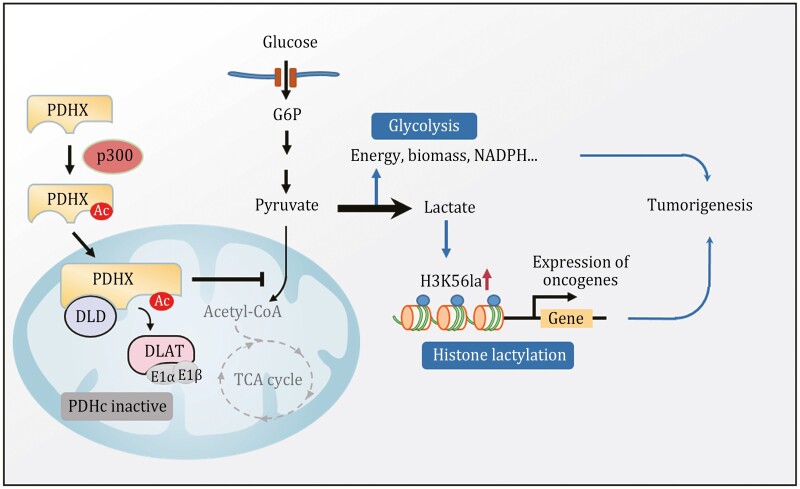
Working model: PDHX acetylation promotes tumor progression by disrupting PDC assembly and activating lactylation-mediated gene expression. Working model shows that PDHX is acetylated by p300 at K488, then impedes the PDC assembly by inhibiting the interaction between PDHX and DLAT, contributing to the aerobic glycolysis and H3K56 lactylation-mediated gene expression, ultimately facilitating tumor progression.

p300, traditionally known for its nuclear localization, also localizes in the cytoplasm and regulates the acetylation of some cytosol proteins (Bian et al, 2017; Son et al, 2024; Su et al, 2017). Furthermore, it has been proposed that p300 may function as a lactyltransferase to catalyze histone lactylation by using lactyl-CoA as a lactyl-donor (Zhang et al, 2019). However, the enzymes that produce lactyl-CoA from lactate in mammalian cells remain unknown, and the levels of lactyl-CoA in tumor cells are extremely low (Varner et al, 2020), which may limit p300’s lactyltransferase activity. In our study, we found p300 can promote the acetylation of the mitochondrial protein PDHX K488 in the cytoplasm, leading to PDC disruption in mitochondria which results in the conversion of glucose to lactate and H3K56 lactylation-mediated gene expression (Fig. 6).

We and others have demonstrated that p300 is highly expressed in HCC (Fig. S2C) (Li et al, 2011; Yokomizo et al, 2011), additionally, the levels of acetyl-coA in tumors are high (Guertin and Wellen, 2023), which leads to high basal levels of PDHX K488 acetylation. The enzymatic activity of p300 and the levels of acetyl-CoA within cells are both regulated by signaling pathways in tumor cells, suggesting that upstream signaling pathway(s) within tumor cells may play a pivotal role in p300-mediated acetylation of PDHX. The high basal levels of PDHX acetylation led to the observation that PDHX-WT and PDHX-K488Q exhibit similar effects on the interaction between PDHX and DLAT (Fig. 3E and 3H), as well as on tumor proliferation (Fig. 5B). Importantly, analysis of clinical HCC samples demonstrated that PDHX Lys 488 acetylation levels greatly increased in cancer tissues compared with the surrounding healthy tissue. Notably, PDHX Lys 488 acetylation levels were positively correlated with the clinical stage of HCC (Fig. 1D–G), suggesting that PDHX Lys 488 acetylation can be exploited as a biomarker and potentially, a therapeutic target for HCC diagnosis and treatment. Collectively, this study uncovers a previously unrecognized regulatory mechanism of PDC assembly and activity, revealing a pro-tumorigenic mechanism that could also serve as a potentially effective therapeutic target in future clinical interventions for HCC.

## Supplementary data

Supplementary data is available at *Protein & Cell* online at https://doi.org/10.1093/procel/pwae052.

pwae052_suppl_Supplementary_Figures_S1-S3

pwae052_suppl_Supplementary_Tables_S1-S7

## Data Availability

The RNA-seq dataset produced in this study are available in the Gene Expression Omnibus: GSE269744.
