# Supplementary material for: PDHX acetylation facilitates tumor progression by disrupting PDC assembly and activating lactylation-mediated gene expression
Source: Protein Cell. 2024 Sep 23;16(1):49–63. doi: 10.1093/procel/pwae052 (PMC11700603; doi:10.1093/procel/pwae052)
Supplement: pwae052_suppl_Supplementary_Figures_S1-S3 [file pwae052_suppl_supplementary_figures_s1-s3.pdf]

Supplementary Information

Supplementary Figure legends

Supplementary Fig 1. PDHX acetylation at Lys 488 is upregulated in HCC and correlated with poor clinical prognosis

(A), Acetylated PDC proteins and their acetylated sites derived from data of nano LC–MS/MS.

(B), IP assay was performed in HEK293T cells transfected with GFP-PDHX. Cells was further treatment with TSA (5 μM, 16 h) and NAM (10 mM, 8 h).

(C), HEK293T cells transfected with Flag-PDHX was further treatment with TSA (5 μM, 16 h) and NAM (10 mM, 8 h). IP was performed using anti-Pan-Ac-k antibody.

(D)-(F), IP assay was performed in HepG2 (**D**), PLC (**E**) or Huh7 (**F**) cells expressing with Flag-PDHX-WT or Flag-PDHX-K488R. Cells were treated with or without TSA (5 μM, 16 h) and NAM (10 mM, 8 h).

(G), A schematic diagram of the PDHX Lys 488 acetylated peptide and non-acetylated peptide used for immunization to generate the anti-Ac-K488 PDHX antibody.

(H), Dot-blot testing of acetylated and non-acetylated peptides using indicated purified antibodies.

(I), Immunoblotting analysis of PDHX Lys 488 acetylation and Flag in HepG2 cells expressing PDHX-WT (WT), PDHX-K488R (K488R) or PDHX-K488Q (K488Q).

Supplementary Fig 2. PDHX Lys 488 is acetylated by p300 in the cytoplasm

(A), RT-qPCR analysis of the p300, CBP, PCAF, HAT1 and GCN5 from HepG2 cells transfected with NTC or shRNA targeting p300, CBP, PCAF, HAT1 and GCN5. Data are presented as the mean ± SD of three independent experiments ($n = 3$). *, $P < 0.05$, compared with NTC group.

(B), Immunoblotting analysis of PDHX Lys 488 acetylation and PDHX in HepG2 cells treated with UMB298 (10 μM), A-485 (10 μM) or C646 (10 μM) for 24 h.

(C), Representative IHC images of p300 staining in normal liver tissue (N) and HCC specimens (T), scale bars, 12.5 μm (upper panel). Statistical quantification of MOD values for p300 staining in IHC assays between normal liver tissues (N) and HCC

specimens (T) (lower panel).

(D), His-HAT and GST-PDHX were purified from *E. coli*, and then pull-down assay was performed.

(E), Immunoblotting analysis of Flag, LaminB1, VDAC and Tubulin in whole-cell lysates (WCL) or mitochondria lysates (Mito) from HepG2 cells expressing with PDHX-WT or PDHX-ΔMTS.

(F), Immunoblotting analysis of Flag-PDHX, LaminB1, VDAC and Tubulin in Whole-cell lysates (WCL), Cytosol lysates (Cyto), Mitochondria lysates (Mito) and Nucleus lysates (Nuc) from HepG2 cells expressing with Flag-PDHX-WT, Flag-PDHX-K488R or Flag-PDHX-K488Q.

Supplementary Fig 3. PDHX Lys 488 acetylation-induced dissociation between PDHX and DLAT inhibits PDC assembly and activity

(A), Glycolysis, Glycolysis Capacity and Glycolysis reserve were analyzed with the ECAR curve. Data are presented as the mean ± SD of three independent experiments ($n = 3$). *, $P < 0.05$, compared with 488R group.

(B), Immunoprecipitation was performed using anti-DLD antibody or IgG in HepG2 cells infected with viruses expressing NTC or shRNA targeting PDHX.

(C), HepG2 cells overexpressing Flag-PDHX-WT or Flag-PDHX-K488Q were treated with C646 for 24 h. Immunoprecipitation was performed using anti-Flag antibody or IgG.

(D), Glycolysis, Glycolysis Capacity and Glycolysis reserve were analyzed with the ECAR curve. Data are presented as the mean ± SD of three independent experiments ($n = 3$). *, $P < 0.05$, between the indicated groups. ns, not significant.

Supplementary Table

Supplementary Table 1. Clinic pathological characteristics of clinical samples and expression profile of PDHX and PDHX K488ac in liver cancer.

Supplementary Table 2. Correlation between PDHX K488ac or PDHX expression and

clinic pathological Characteristics of liver cancer patients.

Supplementary Table 3. Spearman analysis of the correlation between PDHX K488ac or PDHX and clinic pathological characteristics.

Supplementary Table 4. Oligonucleotide sequence of shRNAs.

Supplementary Table 5. Antibody information

Supplementary Table 6. Nucleotide sequences of primers used for qPCR

Supplementary Table 7. Nucleotide sequences of primers used for CUT&tag

Supplementary Fig 1

A

Gene Name	Modification Sites
PDHA1	K83, K321, K344, K386
PDHB	K354
PDHX	K194, K488, K491
DLAT	K466
PDHA2	K180, K242, K385

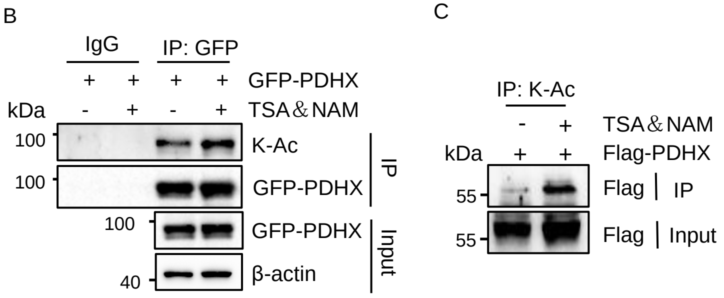

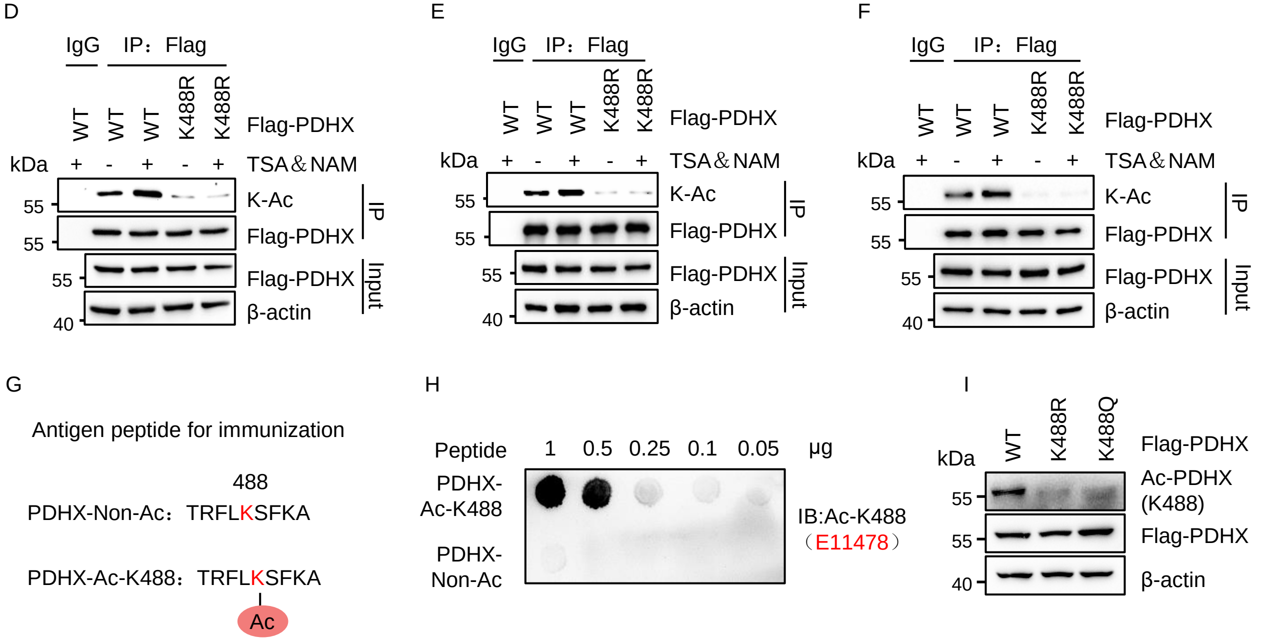

Supplementary Fig 2

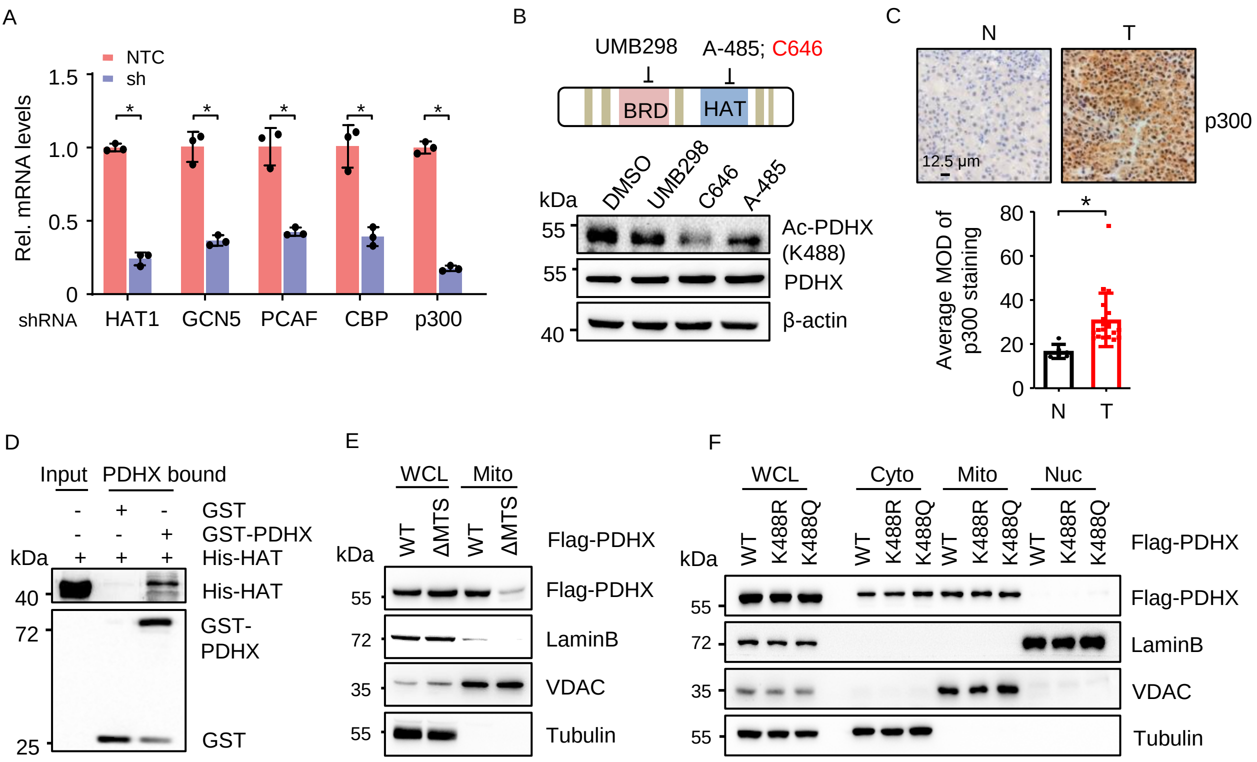

Supplementary Fig 3

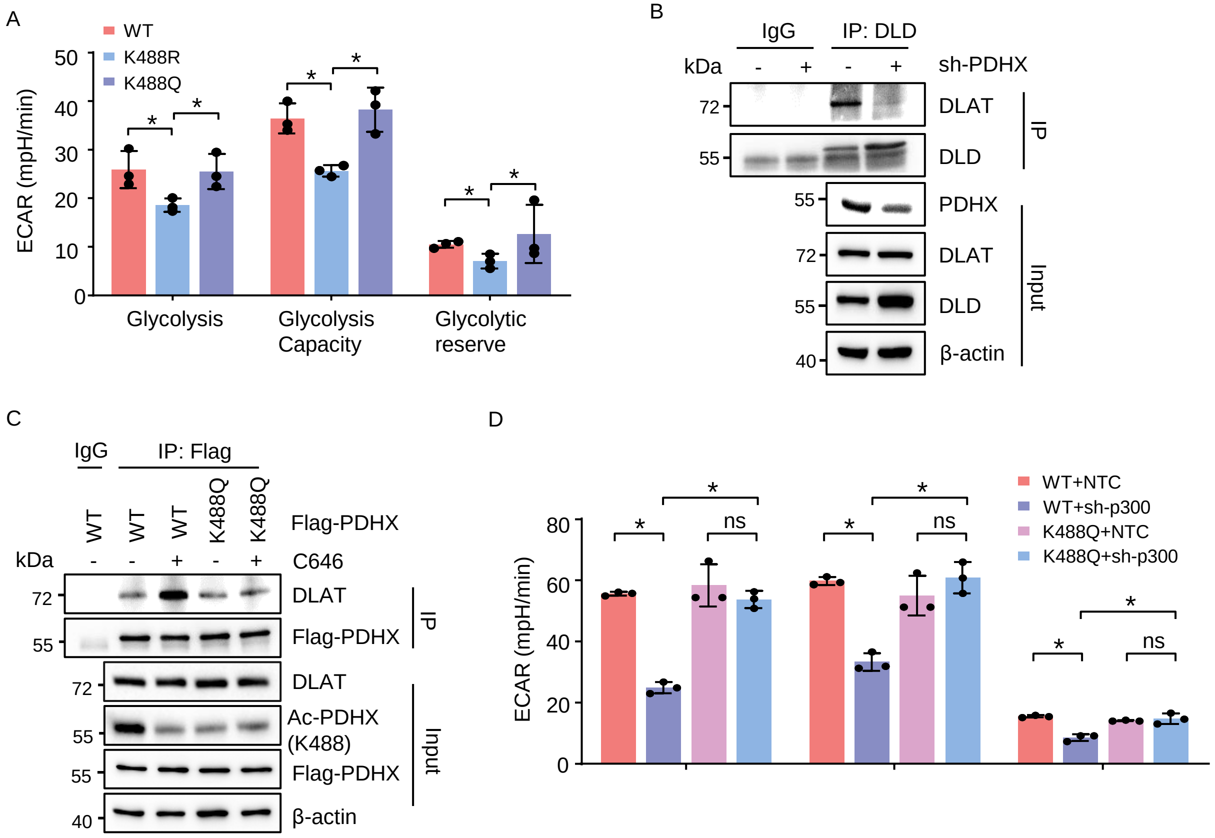

Materials and Methods

Our research complies with all relevant ethical regulations of the University of Science and Technology of China. All animal protocols were approved by the Animal Research Ethics Committee of the University of Science and Technology of China and were performed following the guidelines for use of laboratory animals. The collection and use of clinical materials were approved by the Institutional Research Ethics Committee of the First Affiliated Hospital, University of Science and Technology of China.

Cell culture and reagents

HepG2, HEK293T (RRID: CVCL 4U22), PLC and Huh7 were all obtained from American Type Culture Collection (ATCC) and maintained in DMEM (Gibco, Cat#12800082) supplemented with 10% fetal bovine serum (Vivacell, Cat#C04001) and 1% penicillin–streptomycin (Hyclone, Cat#SV30010). All cells were cultured in a humidified incubator at 37°C and 5%CO_2. Nicotinamide (Sigma, Cat#N0636), Trichostatin A (Sigma, Cat#V900931), C646 (TargetMol, USA, Cat#T2452), A-485 (TargetMol, USA, Cat#T14073), UMB298 (TargetMol, USA, Cat#T9194), Ac-CoA (Sigma, Cat#A2056), DCA (Sigma, Cat#347795).

Plasmids and established stable cells.

All short hairpin RNAs (shRNA) against PDHX, HAT1, GCN5, PCAF, CBP and p300 in PLKO vector were commercially purchased (Sigma-Aldrich). shRNA targeting the PDHX 3′-UTR was constructed in the PLKO vector. shRNAs targeting sequences and information are listed in Table S4. PDHA1, PDHA2, PDHB, DLAT, DLD, PDHX and its mutants, p300 and its domains were subcloned into the pSin-GFP or pSin-3×Flag empty vector. Each lentiviral plasmid was co-transfected with packaging plasmid △8.9 and VSVG into HEK293T packaging cells using PEI (Invitrogen). Viral supernatant was collected 48 hours after transfection, filtered (0.22-nm pore size), and added to HepG2 cells in the presence of 8 mg/mL polybrene (Sigma-Aldrich, Cat#H9268) and stable cells were selected with puromycin (Sigma-Aldrich, Cat#P8833).

Immunoblotting

RIPA buffer (50 mM Tris-HCl pH 8.0, 150 mM NaCl, 5 mM EDTA, 0.1% SDS and 1% NP-40), supplemented with protease inhibitor cocktail (Thermo Fisher Scientific, Cat#A32965), was used to lyse cells on ice for 45 min and protein concentration was determined using a Bradford protein assay kit. Extracted proteins were boiled at 100 °C for 5 min and then subjected to electrophoresis through 6~15% SDS–PAGE. Detailed information for all antibodies used is provided in Table S5.

Immunoprecipitation

For immunoprecipitation assays, briefly, cells were lysed with NP-40 IP buffer (20 mM Tris-HCl (pH 7.5), 150 mM NaCl, 1.5 mM $MgCl_2$, 2 mM EDTA, 1.0% NP-40) supplemented with protease inhibitor cocktail for 2 h on ice, and centrifuged at 12,000g for 10 min at 4°C. The supernatants were incubated with primary antibodies overnight at 4°C and then added protein A/G-Sepharose beads (Thermo Fisher Scientific,

Cat#53133) for further 2 h. The beads were washed four times with 1% NP-40 IP buffer and further boiled in 2 × SDS loading buffer. Protein samples were analyzed by Immunoblotting.

qRT-PCR

According to the manufacturer's instructions, total RNA was extracted from cells or tissues using TRIzol (Life Technologies) and complementary DNA was synthesized from 3 μg of RNA using HiScript III RT SuperMix for qPCR (Vazyme). qPCR was performed using SYBR Green Master Mix (Vazyme). Primer sequences used were shown in Table S6. All samples were normalized to housekeeping genes (18S). The fold change of target mRNA expression was calculated on the basis of the threshold cycle (Ct), where $\triangle Ct = Ct_{target} - Ct_{18S}$ and $\triangle(\triangle Ct) = \triangle Ct_{Control} - \triangle Ct_{Indicated\ condition.}$

Extracellular acidification rate

Measurements of ECAR (Extracellular acidification rate) in HepG2 cells were performed using the Seahorse XF96e analyzer (Seahorse Bioscience, North Billerica, MA, USA) with an XF Glycolysis Stress Test Kit (Agilent, Cat#03020-100). Briefly, 1.5×10^4 cells were seeded per well overnight in a 96-well XF cell culture microplate in growth medium. ECAR was measured with an XF96 analyzer in XF base medium containing 2 mM glutamine (pH 7.4) following sequential additions of glucose (10 μM), oligomycin (1 μM) and 2-DG (50 mM). Data were analyzed by a Seahorse XF Glycolysis Stress Test Report Generator.

Expression and purification of recombinant proteins

The PET-22b-HAT were transfected into *Escherichia coli* Rosetta (DE3) (Tsingke Biological Technology, China), and the expression of the His-HAT protein was induced with IPTG. The bacterial cells were collected, and the proteins were subjected to by ultrasonic lysis. The His-HAT protein was purified by BeaverBeads™ His-tag protein Purification Kit (BEAVER Nano-Technologies Co., Ltd, SuZhou, China). PGEX-4T1-EV, PGEX-4T1-PDHX or PDHX's mutants were transfected into *E. coli* Rosetta (DE3). IPTG was used to induce the expression of GST-tagged proteins. The bacterial cells were collected and broken by ultrasonic wave. Then glutathione Sepharose 4B (GE life, Cat#17075601) were used to adsorb the GST-tagged proteins. Then PBST (137 mM NaCl, 2.7 mM KCl, 10 mM Na_2HPO_4, 2 mM KH_2PO4, 1% Tween 20) buffer was used to rinse and purify the GST-tagged proteins and followed by eluted with an elution buffer containing 50 mM Tris-HCl (pH 8.0) and 20 mM reduced glutathione (GSH).

GST pull-down experiment

Purified GST tagged proteins and His-tag proteins were used for pull-down experiments in pull-down buffer (PDB) (150 mM NaCl, 50 mM Tris (pH 7.5), 0.1% NP-40, 5 mM DTT). After incubation, the beads were pelleted and washed with PDB buffer followed by elution of proteins and analysis by Immunoblotting.

***In vitro* acetylation assay**

Purified various GST-PDHX mutants were acetylated by recombinant His-HAT. Briefly,

5 μg GST-PDHX or mutants were incubated with 1 μg p300-HAT domain in HAT buffer (50 mM Tris-HCl, pH 8.0, 50 mM KCl, 5% glycerol, 0.1 mM EDTA, 1 mM DTT, 2 μM TSA, 50 μM Ac-CoA sodium salt and 1 mM PMSF) at 30°C for 1 h. The reaction was stopped by adding 5×SDS loading buffer and by boiling the samples for 5 min. And products were subjects to SDS-PAGE analysis and immunoblotted with the indicated antibodies.

Verification of PDHX acetylation by HPLC-MS/MS

The supernatants were incubated with primary antibodies overnight at 4 °C and then added protein A/G-Sepharose beads (Thermo Fisher Scientific, Cat#53133) for further 2 h. The beads were washed seven times with 1 mL 50 mM NH_4HCO_3, followed by digesting in 50 μL 50 mM NH_4HCO_3 with Glu-C at 37°C for 16 h. The Glu-C-digested peptides were desalinated using a desalting column and then vacuum-dried. The peptides were resuspended with solvent A (A: water with 0.1% formic acid) and analyzed by HPLC-MS/MS.

PDH Activity Assay

The activity of the PDC was measured using the Pyruvate Dehydrogenase Enzyme Activity Microplate Assay Kit (abcam, Cat#ab109902) according to manufacturer's instructions. All measurements were performed in at least three biological replicates.

Measurement of lactate production

The extracellular lactate was measured using the cell culture medium with lactate assay kit (BioVision, Cat#K627/ab65331) according to the manufacturer's instruction. The values were normalized to the protein concentration. All measurements were performed in at least three biological replicates.

RNA-seq analysis

Total RNA was extracted using TRIzol Reagent (Life Technologies) following the manufacturer's instructions. RNA integrity was assessed by RNA integrity number and determined using an Agilent 2100 Bioanalyzer. A total amount of 3 μg of RNA per sample was used for analysis. Sequencing sampling was performed from one single replicate. Libraries were generated using a NEBNext Ultra RNA Library Prep Kit for Illumina (NEB). RNA-seq was performed on an Illumina NovaSeq 6000 platform by Novogene (Tianjin). Reads were aligned to the human genome hg19. TopHat2 v.2.1.0 and cufflinks v.2.2.1 were used to analyze RNA-seq data. Gene differential expression analysis was carried out with the DEGSeq R package (1.26.0). The RNA-seq data reported here have been deposited in the NCBI Gene Expression Omnibus (GEO, RRID: SCR_005012).

CUT&Tag Assays

HepG2 cells were grown to suitable density in 60-mm dishes, and used for CUT&Tag assay analysis. A Hyperactive Universal CUT&Tag Assay Kit (Vazyme, Cat#TD903-01) was used following the manufacturer's recommended protocol. And anti-H3K56la antibody (PTM BioLab, Cat#PTM-1421RM) was used to pull down DNA-protein

complexes, and rabbit IgG was used as control. Purified DNA was quantified by qRT-PCR. Primer sequences used were shown in Table S7.

Clinical human HCC specimens

Snap-frozen HCC tissues and corresponding noncancerous tissues that were at least 2 cm distant from the edge of tumors were collected from 11 patients with HCC in the First Affiliated Hospital of University of Science and Technology of China (Hefei, China). Total RNA and protein were extracted from paired HCC and noncancerous tissues and then detected by qPCR and immunoblot, respectively. Formalin-fixed, paraffin-embedded primary HCC specimens from 118 patients were randomly selected from the archives of the First Affiliated Hospital of University of Science and Technology of China. Clinical data and pathological characteristics of patients, including age, gender, tumor size, tumor lymph nodes, serum alpha-fetoprotein, hepatitis B surface antigen, vascular invasion and liver cirrhosis, were gathered retrospectively from patient medical records. Detailed patient data are shown in Table S1-3. The clinical staging of tumors was defined by the fifth edition of the American Joint Committee on Cancer/International Anti-Cancer Alliance TNM Classification System. To use these clinical materials for research purposes, both the patients' previously obtained written informed consent and the research approval of the Institutional Research Ethics Committee of the First Affiliated Hospital of University of Science and Technology of China were obtained. All patients volunteered and received no compensation. The studies were conducted in accordance with ethical guidelines of the Declaration of Helsinki.

IHC staining

Immunohistochemistry was performed as previously described. In brief, samples were dewaxed using xylene and rehydrated with graded ethanol. After antigen retrieval, sections were incubated in 0.3% hydrogen peroxide for 10 min to block endogenous peroxidase activity. Sections were then pre-incubated in normal goat serum for 15 min to prevent nonspecific staining. Samples were then incubated with anti-PDHX or PDHX Lys 488 acetylation antibodies at room temperature. Four to six hours later, a secondary antibody was used followed by incubation with DAB Chromogen dilution solution. The IHC staining was quantitatively analyzed using TissueFAXS (TissueGnostic) and HistoQuest Rel.4.0 (TissueGnostic, RRID:SCR_014823). The mean optical density (MOD) was verified through analysis of ten staining fields of each section, and the differences between groups were compared by t-test.

Animal studies

All animals were housed at a suitable temperature (22~24°C) and humidity (40~70%) under a 12/12-h light/dark cycle with unrestricted access to food and water for the duration of the experiment. All animal studies were approved by the Animal Research Ethics Committee of the University of Science and Technology of China. For xenograft experiments, 5×10^6 HepG2 cells stably overexpressing PDHX-WT, PDHX-K488R or PDHX-K488Q. For the *in vivo* xenograft experiment with DCA (Sigma, Cat#347795) treatment, mice were treated with DCA (156 mg per kg (body weight)) or PBS by injection every 3 days. We used a digital caliper to measure tumor volume every 3 days,

and the following formula to calculate tumor volume: length (mm)×width (mm)×depth (mm)× 0.52. The xenograft tumor burden was less than the maximum tumor size (1 cm^3) approved by the Animal Research Ethics Committee of the University of Science and Technology of China. *In vivo* experiments were randomized to distribute tumor volume among different treatment conditions at the time of treatment initiation. The study is compliant with all ethical regulations of animal research.

Statistical analysis

The data are presented as either mean ± SD or mean ± SEM of at least three independent experiments. Statistical analyses were performed using Prism 8 (GraphPad Prism Software (RRID:SCR_002798)). Group differences ($P < 0.05$, *) were analyzed by two-tailed Student's t-test or one-way ANOVA. Kaplan–Meier curves were used to depict survival function from lifetime data for human patients using the log-rank test. No statistical methods were used to predetermine sample size, but ours are similar to those reported in previous publications. No data were excluded from the analyses. The experiments were not randomized, except that mice were randomly grouped before different treatments. Data collection and analysis were not performed blind to the conditions of the experiments, except for IHC score analysis.
